# Association between historical lead exposure, population density, and autism prevalence: a county-level ecological study in Norway

**DOI:** 10.3389/fnins.2026.1729731

**Published:** 2026-02-04

**Authors:** Aaron Grossman

**Affiliations:** Independent Researcher, Miami, FL, United States

**Keywords:** autism spectrum disorder, ecological study, environmental epidemiology, metal toxicity, neurodevelopment, population density, lead

## Abstract

**Background:**

Autism spectrum disorder (ASD) prevalence has increased markedly across high-income countries, including Norway, yet environmental drivers remain poorly understood. We hypothesized that historical lead exposure may inversely associate with ASD risk and potentially suppress the effects of vehicle emissions associated with urbanicity.

**Methods:**

We conducted a county-level ecological study using ASD prevalence data from the Norwegian Patient Register for children aged 6–12 years (born 1999–2005). Mean dental lead concentrations, measured in deciduous teeth collected between 1990 and 1994 (*n* = 2,746), served as a proxy for early-life lead exposure. Population density in 2002 was used as a proxy for vehicle emissions associated with urbanicity. ADHD and cerebral palsy (CP) were included as negative controls. Associations with log-transformed ASD prevalence were assessed using multivariable linear regression. Sensitivity analyses examined a later birth cohort and excluded an outlier county.

**Results:**

Dental lead levels were inversely associated with ASD prevalence (*p* < 0.01), while population density was not significantly associated until adjusted for lead exposure (*p* = 0.08 and *p* < 0.05 with outlier excluded), consistent with a suppressor effect or negative confounding. No associations were observed for ADHD or CP. In sensitivity analyses, lead remained inversely associated with ASD only in multivariable models, while population density became a significant predictor.

**Conclusion:**

Higher historical lead exposure was associated with lower ASD prevalence in Norway. Declining lead exposure may have unmasked the neurodevelopmental effects of vehicle emissions. These findings support further investigation into metal interactions and highlight the complexity of environmental influences on ASD risk.

## Background

Autism spectrum disorder (ASD) is a neurodevelopment disorder that manifests in early childhood. The reported prevalence of ASD has been increasing steadily over the last several decades, rising in the US from 4 per 10,000 in 1962–1967 (infantile autism) to 280 per 10,000 in 2023 (Maenner et al., 2023; Treffert, 1970). Whether this remarkable increase represents a true epidemic or reflects enhanced awareness remains debated.

Global disparities in ASD prevalence further complicate our understanding of its epidemiology. Even studies from similar time periods have reported vastly different prevalence rates: for instance, 1 per 10,000 in children aged 0–14 years in Oman compared to 260 per 10,000 in children aged 7–12 years in Goyang City, South Korea (Al-Farsi et al., 2011; Kim et al., 2011). Due to such discrepancies, some researchers have sought an environmentally-based rationale to understand the etiology of ASD (Nevison, 2014). Though researchers have studied potential links between metal exposure and ASD, studies have been inconsistent (Rahbar et al., 2021). None have satisfactorily explained the onset, persistence, and continued expansion of measured prevalence, nor have they addressed the observed geographical disparities.

However, metals exposure remains a key area of interest. Notably, Schmidt et al. (2014) reported that maternal iron (Fe) supplementation during pregnancy was associated with a reduced risk of ASD in offspring, with an apparent dose-response. We have previously suggested that the long-term decline in iron deficiency among infants, women of child-bearing age, and pregnant women makes it unlikely that mitigating a putative maternal iron deficiency would explain the findings (Grossman, 2017). Instead, we proposed that iron may have been acting as an effect modifier, reducing the uptake and exposure to a potentially toxic metal (Grossman, 2017).

This hypothesis raises the possibility that metals with similar properties to iron might also reduce ASD risk. Counterintuitively, a steep decline in exposure to lead (Pb) over the past few decades, driven by concerns over neurodevelopmental impairment, may have inadvertently contributed to the dramatic rise of ASD. Decreased maternal lead exposure may have increased ASD risk by enabling higher absorption or bioavailability of other environmental metals linked to neurodevelopmental harm in a manner analogous to iron (Meltzer et al., 2010). Variations in lead exposure due to differing national and local regulations and pollution sources could explain much of the temporal and geographic discrepancies in ASD prevalence.

With this understanding, we present a county-level ecological analysis in Norway examining whether higher lead exposure, inferred from lead content in deciduous teeth collected in the early 1990s, is associated with lower ASD prevalence in children born a decade later.

## Materials and methods

County-level ASD prevalence (ICD-10 code F84, Pervasive developmental disorders) among children aged 6–12 years was extracted from a national study by Surén et al. (2013) using the Norwegian Patient Register for years 2008–2011 (children were born from 1999 to 2005). ADHD (ICD-10 F90) and cerebral palsy (CP, ICD-10 G80) prevalence was extracted from the same study as comparator outcomes (Surén et al., 2013). CP served as a negative control due to its largely non-environmental etiology; ADHD, on the other hand, while partially linked to toxicants, served to assess specificity given overlapping diagnostic practices.

Geometric mean (GM) concentration of lead (Pb) was sourced from a study by Tvinnereim et al. (1997) in which the authors measured the dental content of this metal from deciduous teeth collected by local dentists from the Norwegian Public Dental Health Service from 1990 to 1994 in all counties of Norway. Full methods involving analyzation of lead content have been previously published (Tvinnereim et al., 1997). We hypothesized that higher county-level lead concentrations would be associated with lower ASD prevalence, potentially by reducing exposure to unknown ASD-causing toxic metals (see Supplementary Figure 1).

Although the teeth used to measure metal levels were collected 5–15 years prior to the birth of the study cohort, we assumed that the relative geographic distribution of lead exposure remained stable across counties over the elapsed period. While absolute lead levels declined nationally—evidenced by the drop in dental lead geometric means from 2.65 μg/g in the 1970s to 1.27 μg/g in the 1990s (Tvinnereim et al., 1997)—and likely continued to fall throughout the capture of the entire birth cohort, relative exposure levels across counties were likely preserved. This assumption is supported by the simultaneous nationwide phase-out of leaded gasoline in 1995, which removed the primary source of atmospheric lead across all counties (Benestad, 2005). Given that other atmospheric sources were eliminated previously and drinking water contributes only trivial exposure (Tvinnereim et al., 1997), subsequent exposure was likely driven by stable legacy soil contamination (Mielke et al., 2022). To test the robustness of this assumption, we investigated the temporal correlation between lead concentrations in moss (*Hylocomium splendens*) between 1990 and 2000 across 22 locations in 15 counties, based on estimates from Steinnes et al. (2005). Although moss primarily reflects atmospheric deposition mainly originating from sources in other regions of Europe, Tvinnereim et al. found it to be the strongest predictor of dental lead concentrations. Our analysis yielded an r-squared value of 0.62, indicating a high degree of consistency in spatial distribution of lead exposure over time and supporting the use of historical dental lead as a proxy for the study cohort’s environment.

Studies have shown that living near roadways with a high concentration of vehicle traffic is likely to expose one to high concentrations of traffic-related air pollution including fine particulate matter at 2.5 microns or less in diameter (PM_2.5_) (Antonczak et al., 2023; Jia et al., 2021). Other studies have shown a more direct association between population density and PM_2.5_ emissions from vehicles (Borck and Schrauth, 2021; Han and Sun, 2019; Wrightson et al., 2025). Therefore, in the current study, population density served as a proxy for exposure to environmental toxicants caused by vehicular emissions. It was estimated for the year 2002 (midpoint of birth cohort) by calculating the weighted average of municipality-level population densities within each county, with weights proportional to municipal population size (supplied by Statistics Norway). Although population density may affect access to mental health resources, our main univariate analysis found no relationship between population density and ASD prevalence (*p* = 0.56). Further, every municipality in Norway must offer a child health clinic, providing universal developmental and health services to children from birth through adolescence (The Norwegian Directorate of Health, 2019).

County-level median income (in Norwegian kroner) per couple with children in 2002 was obtained as an indicator of socioeconomic status (Statistics Norway). The total number of persons per psychiatric unit was calculated by dividing total population by number of psychiatric units (sourced from the Surén et al. (2013) study); this served as a proxy for psychiatric service availability.

### Statistical analysis

All prevalence outcomes were log-transformed to reduce skew and normalize distributions. We used simple and multivariable linear regression to assess associations between log-prevalence of neurodevelopmental disorders and lead exposure, adjusted for log-transformed population density. Though we initially included income and psychiatric institution density in exploratory models to assess their independent contributions, neither variable was statistically significant in the overall model (both *p* > 0.80), and their inclusion did not materially change the effect estimate for lead. Inclusion of income and psychiatric density resulted in a negligible (< 1%) change in the lead coefficient (−0.988 to −0.981). Nested model comparisons via ANOVA confirmed that these variables did not significantly improve model fit (income: *F* = 3.63, *p* = 0.04; psychiatric density: *F* = 3.65, *p* = 0.04; both: *F* = 2.56, *p* > 0.08 versus base model: *F* = 5.75, *p* = 0.01). Thus, to preserve statistical power and avoid overfitting in this small-sample (*n* = 19 counties) ecological analysis, both variables were excluded from the final model.

To identify influential observations, we calculated standardized residuals, Cook’s distance, and leverage values. Telemark county was identified as an outlier with a standardized residual of 2.64, exceeding the 2.5 threshold. While Oslo and Vest-Agder exhibited high leverage, their low residuals (both < | 0.9|) and low Cook’s distance (< 0.2) confirmed they were consistent with the overall model trend. Visual inspection of added-variable plots confirmed Telemark county’s deviation (see Supplementary Figure 2).

We conducted a series of sensitivity analyses. First, Telemark county was identified as a potential outlier based on the above analysis and excluded. Second, ASD prevalence of children aged 2–8 years (born between 2006 and 2014) from a follow-up study by Surén et al. (2019), was extracted, and analyses conducted with dental lead and population density covariates. This allowed us to assess whether observed associations between lead and ASD persisted in a temporally distinct cohort, despite the lag between exposure and outcome measurement. Finally, we conducted sensitivity analyses to assess whether variation in sample representativeness affected our results. The representativeness of deciduous teeth varied substantially across counties (0.02%–0.31% of the population). To evaluate whether counties with smaller samples (and potentially less precise lead measurements) influenced findings, we first examined the correlation between sampling proportion and the geometric standard deviation of dental lead within each county; no significant correlation was observed (*r* = −0.13, *p* = 0.59), suggesting measurement precision was not systematically related to sample size but more likely intra-county variance. We then scaled the county-level geometric mean dental lead concentration by one geometric standard deviation in each direction to simulate scenarios where exposure was higher or lower than measured—effectively testing whether results were sensitive to measurement error that might be more pronounced in poorly sampled counties. These analyses demonstrated that the observed associations remained significant across both scenarios, indicating that findings were robust to potential measurement variability and were not driven by extreme values from counties with fewer samples or wider standard deviations. We also conducted correlation analysis of regression residuals against teeth representativeness and found no significant correlation (*r* = 0.49, *p* = 0.26).

No missing data were present for any variables included in the primary analysis. Variance inflation factors (VIFs) were calculated to assess multicollinearity and were below 2.0 in all models. All regression analyses were conducted with SAS^®^ OnDemand for Academics. Given the ecological design and small sample size, findings should be interpreted with caution and viewed as hypothesis-generating.

This analysis used publicly available, aggregated data and did not require institutional review board approval.

## Results

A total of 19 Norwegian counties were included in the analysis, which is more than the current 15 counties that Norway maintains after several mergers (Table 1). Prevalence of ASD ranged from 0.27% to 1.46% (mean: 0.61%, SD 0.27%), ADHD from 1.07% to 3.46% (mean: 2.12%, SD: 0.67%), and CP from 0.17% to 0.50% (mean: 0.30%, SD: 0.07%). Geometric mean dental lead ranged from 1.03 to 1.91 μg/g (mean: 1.29 μg/g, SD: 0.26). Sample sizes ranged from 39 to 393 teeth per county, representing 0.02% to 0.31% of each county’s population. Population density ranged from 4 to 1,203 persons/km^2^, income from 457,600 to 539,200 NOK per couple, and persons per psychiatric institution from 21,799 to 102,945. The correlation between dental lead and population density was 0.46 (*p* < 0.05, Supplementary Table 1).

**TABLE 1:** Prevalence of neurodevelopmental conditions, environmental factors, and socioeconomic indicators by Norwegian county.

				Deciduous teeth (1990–1994)				
County	ASD, 2008–2011	ADHD, 2008–2011	CP, 2008–2011	*n*, % of population^a^	Pb, μ g/g^b^ (SD)	Population density, 2002 (persons/sq. km)	Yearly income per couple (NOK, 2002)	Persons / psychiatric institutions (2008–2011)
Akershus	0.49%	1.37%	0.34%	158, 0.03%	1.31 (1.84)	321	539,200	43,393
Aust-Agder	0.31%	3.46%	0.29%	108, 0.10%	1.44 (2.01)	81	465,400	102,945
Buskerud	0.53%	1.99%	0.30%	244, 0.10%	1.09 (1.85)	251	487,600	21,799
Finnmark	0.47%	2.39%	0.28%	230, 0.31%	1.27 (1.77)	4	495,600	18,433
Hedmark	0.46%	3.06%	0.31%	53, 0.03%	1.03 (1.45)	25	458,200	37,593
Hordaland	0.54%	1.78%	0.25%	393, 0.09%	1.48 (1.82)	329	491,200	48,695
More and Romsdal	0.70%	1.98%	0.26%	200, 0.08%	1.23 (1.82)	214	485,900	60,964
Nordland	0.49%	3.32%	0.33%	79, 0.03%	1.61 (1.61)	16	474,400	33,929
Nord-Trondelag	0.51%	1.74%	0.22%	39, 0.03%	1.12 (2.06)	16	457,600	63,729
Oppland	0.65%	1.72%	0.24%	85, 0.05%	1.17 (2.01)	26	460,800	91,618
Oslo	0.50%	1.12%	0.29%	98, 0.02%	1.87 (1.94)	1,203	509,100	51,259
Ostfold	0.68%	1.90%	0.17%	60, 0.02%	1.13 (1.98)	221	465,000	42,124
Rogaland	1.04%	2.55%	0.35%	106, 0.03%	1.06 (1.76)	636	502,600	38,138
Sogn and Fjordane	0.56%	1.32%	0.37%	150, 0.14%	1.03 (1.73)	10	486,500	35,760
Sor-Trondelag	0.44%	2.39%	0.50%	130, 0.05%	1.39 (1.85)	321	476,800	38,046
Telemark^c^	1.46%	2.14%	0.27%	94, 0.06%	1.12 (1.76)	72	471,400	41,428
Troms	0.55%	2.41%	0.32%	172, 0.11%	1.08 (1.76)	22	485,000	30,335
Vest-Agder	0.27%	1.07%	0.31%	95, 0.06%	1.91 (2.00)	209	466,300	39,463
Vestfold	0.95%	2.48%	0.28%	119, 0.05%	1.19 (1.70)	315	485,200	54,114

*^a^*% of population represents the proportion of county population sampled for dental lead analysis, indicating representativeness of exposure measurement.

*^b^*Geometric mean.

*^c^*Telemark was removed as an outlier in sensitivity analyses. ASD, autism spectrum disorder; ADHD, attention deficit hyperactivity disorder; CP, cerebral palsy; NOK, Norwegian Krone; Lead, dental lead concentration.

**FIGURE 1:**
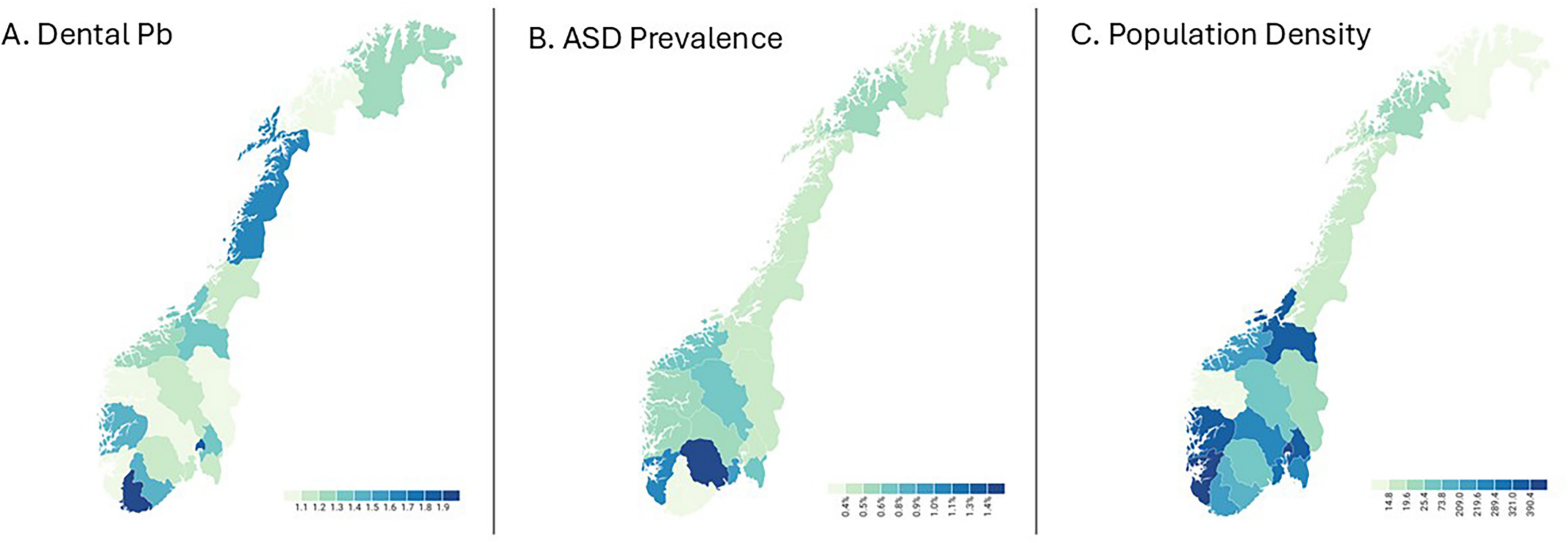
Choropleth maps of Norwegian counties showing **(A)** mean dental lead (Pb) concentration in deciduous teeth (μg/g), **(B)** autism spectrum disorder (ASD) prevalence (%), and **(C)** population density (persons per km^2^). Lead levels were derived from tooth samples collected between 1990 and 1994; ASD prevalence reflects diagnoses from 2008 to 2011 in children aged 6–12 years. Population density is from the year 2002, the midpoint of the birth cohort. Darker shades represent higher values. ASD prevalence and population density are both highest in southern counties, while dental lead shows no consistent geographic pattern.

**FIGURE 2:**
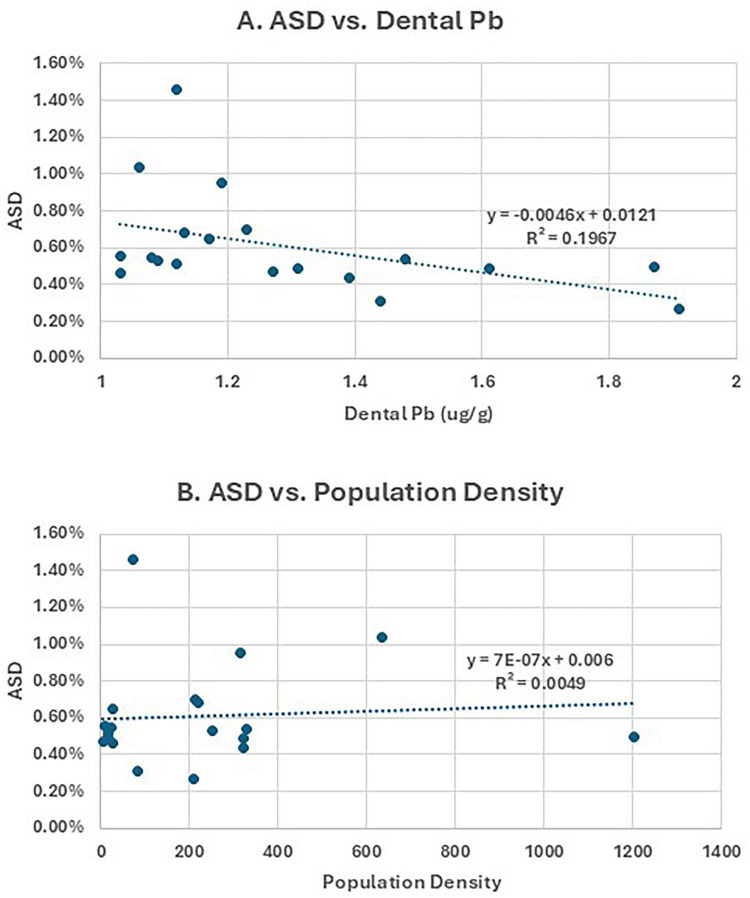
Scatter plots of **(A)** ASD prevalence (%) vs. mean dental lead (Pb) concentration in deciduous teeth (μg/g) and **(B)** ASD prevalence (%) vs. population density (persons per km^2^).

Notably, the county of Vest-Agder had both the highest mean dental lead concentration and the lowest prevalence of ASD and ADHD. Figure 1 displays choropleth maps illustrating regional variation in ASD prevalence, population density, and dental lead levels. While there is no clear geographic pattern in the distribution of lead across counties, population density is highest in the southern regions of Norway, where ASD prevalence also tends to be elevated. This spatial overlap raises the possibility that vehicle emission-related exposures may contribute to regional differences in ASD prevalence, independent of lead. Plots of ASD versus dental lead and ASD versus population density are found in Figure 2.

In simple linear regression, dental lead levels were inversely associated with log-transformed ASD prevalence (*p* = 0.01) (Table 2). No significant association was found between log-transformed population density and log-transformed ASD (*p* = 0.56). Models using ADHD or CP as outcomes did not show significant associations, supporting the specificity of the lead–ASD relationship.

**TABLE 2:** Univariate linear regression analysis of natural log-transformed neurodevelopmental disorder prevalence by environmental/socioeconomic covariates at county level.

Model	Variable	ln(Density)	Dental lead	Income	Persons / psychiatric institutions
ln(ASD)	Intercept	−5.3364	−4.1465	−6.1776	−5.0892
	Coefficient	0.0353	−0.7981	2.07E-06	−1.86E-06
	SE	0.059	0.2996	4.66E-06	4.46E-06
	Adj. R-Sq.	−0.037	0.2529	−0.0466	−0.0481
	*p*-value	0.5574	0.0164	0.6615	0.6818
ln(ADHD)	Intercept	−3.6414	−3.3271	−1.3584	−3.9576
	Coefficient	−0.059	−0.45	−5.29E-06	1.05E-06
	SE	0.0493	0.2872	3.82E-06	3.86E-06
	Adj. R-Sq.	0.0235	0.0748	0.0482	−0.0542
	*p*-value	0.2477	0.1356	0.1848	0.7878
ln(CP)	Intercept	−5.8835	−5.9895	−7.6048	−5.6894
	Coefficient	0.0101	0.1175	3.66E-06	−3.16E-06
	SE	0.0335	0.1989	2.48E-06	2.41E-06
	Adj. R-Sq.	−0.0532	−0.0375	0.0613	0.0383
	*p*-value	0.7666	0.5626	0.1585	0.2076

Lead, dental lead concentration; Density, population density; CP, cerebral palsy.

In multivariable regression models (Table 3) adjusted for log-transformed population density, higher dental lead was significantly associated with lower ASD prevalence (*p* = 0.005), while population density was positively but non-significantly associated with ASD (*p* = 0.08). The model explained 42% of the variance in ASD prevalence (*R*^2^ = 0.42; adjusted *R*^2^ = 0.35, *p* = 0.01). We tested for interaction between dental Pb and log-transformed population density, but the interaction term was not statistically significant (*p* = 0.72). Sensitivity analyses scaling dental lead levels by one geometric standard deviation in each direction confirmed the robustness of findings (Supplementary Table 2). Both higher (+1 STD: *p* = 0.002, *R*^2^ = 0.39) and lower (−1 STD: *p* = 0.03, *R*^2^ = 0.18) lead exposure scenarios maintained significant inverse associations with ASD prevalence, indicating results were not driven by sampling variability in counties with fewer dental specimens.

**TABLE 3:** Multiple linear regression analysis of natural log-transformed neurodevelopmental disorder prevalence by dental lead at county level.

Model	Variable	Coefficient	SE	*p*-value
ASD	ln(Density)	0.0921	0.0499	0.0836
	Lead	−0.9877	0.2987	**0.0045**
	Model Adj. R-Sq.	0.3455 (*p* = **0.0131**)		
ADHD	ln(Density)	−0.0376	0.0519	0.4788
	Lead	−0.3726	0.3102	0.2472
	Model Adj. R-Sq.	0.0482 (*p* = 0.2624)		
CP	ln(Density)	0.0038	0.0365	0.9185
	Lead	0.1097	0.2183	0.6223
	Model Adj. R-Sq.	−0.1016 (0.8455)		

Bold *p*-values indicate statistical significance at α = 0.05. lead = dental lead concentration; CP, cerebral palsy. Each model includes ln population density as a covariate along with dental lead concentrations.

Excluding the outlier county of Telemark strengthened the results: both lead (*p* = 0.002) and population density (*p* = 0.04) were significant, and the model explained 50% of the variance (adjusted *R*^2^ = 0.43). The significance of population density only after adjusting for Pb supports a potential modifying role.

In sensitivity analyses of ASD prevalence of children born in 2006–2014, log-transformed population density was significantly associated (*p* = 0.0012) while dental lead remained inversely associated (*p* = 0.01) with ASD (Table 4). The model explains 53% of the variance in ASD prevalence (adjusted *R*^2^ = 0.47, *p* = 0.002). Notably, with univariate analyses, population density was significantly associated with ASD (*p* = 0.02), however, lead was not (*p* = 0.24). This temporal pattern supports our hypothesis that declining lead exposure unmasked urban-related risks: in the earlier birth cohort (1999–2005), lead was protective but population density showed no effect; in the later cohort (2006–2014), population density became a significant univariate predictor while lead’s protective effect weakened.

**TABLE 4:** Sensitivity analysis using ASD prevalence in children born 2006–2014 (*N* = 19 counties).

Model	Variable	Coefficient	SE	*p*-value	Adj. R-Sq.
Univariate Multivariate	ln(Density)	0.00064	0.00025	**0.0192**	0.2402
	Lead	−0.00206	0.00168	0.2374	0.027
	ln(Density)	0.00086	0.00022	**0.0012**	
	Lead	−0.00383	0.00132	**0.0103**	
	Model Adj. R-Sq.	0.4718 (*p* = **0.0024)**			

Lead, mean dental lead content (μg/g); ln(Density), natural log of persons/km^2^. Bold *p*-values indicate statistical significance at α = 0.05.

## Discussion

This ecological analysis found no significant association between population density and ASD prevalence in simple regression. However, dental lead levels were significantly and inversely associated with ASD prevalence. When the outlier county of Telemark was excluded, population density became significantly associated with ASD, but only after adjusting for lead levels. This emergence of significant association between population density and ASD after adjusting for lead is consistent with a suppressor effect or negative confounding—where lead accounts for variance shared with population density that was previously masking its true association with ASD. While the study is limited by its small sample size (*n* = 18–19 counties), it highlights a previously unreported interplay between lead exposure, population density (a proxy for vehicle-related emissions), and ASD prevalence.

Interestingly, in sensitivity analyses using ASD prevalence data from a later birth cohort (2006–2014), population density was significantly associated with ASD in univariate models, while dental lead was no longer inversely associated—though the inverse association with lead persisted in multivariable models. This divergence may reflect two possibilities. First, the assumption of stable lead exposure across counties (i.e., relative to each other) may not hold over time; dental lead levels measured from teeth collected in the early 1990s may no longer accurately represent exposure conditions for children born more than a decade later. Second, as environmental lead levels declined—due to regulatory removal from gasoline, paint, and other sources—other urban-associated exposures linked to population density (e.g., vehicle emissions) may have become more prominent determinants of ASD risk. These findings suggest that the apparent protective association with lead may have attenuated over time, allowing the effects of urban-related exposures to emerge more clearly in more recent cohorts. Together—limitations of the study notwithstanding—the results depict ASD to be a heavily environmentally influenced disorder.

Telemark exhibited unusually high ASD prevalence and was excluded as an outlier in sensitivity analyses. One plausible explanation is the presence of Herøya Industrial Park within the town of Porsgrunn and the larger Grenland region, where historical industrial emissions may have influenced local environmental exposures. Recent remediation efforts have focused on covering contaminated sediments to prevent the spread of legacy pollutants (Herøya Industripark AS, 2023). Notably, Telemark was not an outlier in sensitivity analyses of ASD prevalence among children born between 2006 and 2014, potentially indicating reduced emissions following the implementation of earlier environmental controls. While this interpretation is speculative, it underscores the potential for regional variation in ASD risk driven by localized environmental factors.

### Historical trends

Norway, much like the rest of the developed world, has experienced a steep decline in lead exposure due to the phase-out of leaded gasoline as well as other successful environmental remediation efforts (Tvinnereim et al., 1997). For instance, mean lead levels in deciduous teeth in Norway fell from 2.92 μg/g in the 1970s to 1.27 μg/g in the early 1990s (Tvinnereim et al., 1997). Simultaneously, ASD prevalence in Norway has increased by an order of magnitude from children born in the 1970s to those born in the early 2000s (Surén et al., 2013).

Global trends in reduced lead exposure, particularly with respect to the phase-out of leaded gasoline, aligns with worldwide variations in ASD prevalence. Nations which had banned leaded gasoline early, such as the US, Japan, Canada, South Korea, Sweden, and the UK, reported ASD prevalence exceeding 100 per 10,000 by the early 2010s (Lovei, 1998; Zeidan et al., 2022). Conversely, a culturally diverse group of nations with historically higher lead exposure due to delayed bans or ongoing industrial pollution have tended to report lower ASD prevalence: for example, Bangladesh, Ecuador, Israel, Oman, and Venezuela (Zeidan et al., 2022). While these geographic associations are intriguing, they should be interpreted cautiously given the potential for diagnostic, cultural, and health system differences.

### Potential confounders

Previous research suggests substantial portions of historical increases in ASD were due to diagnostic shifts; for instance, one-third of the 1980–1991 prevalence increase in Denmark (Hansen et al., 2015). However, these factors do not explain prevalence increases during periods of stable diagnostic criteria nor do they explain regional differences in ASD prevalence, such as observed in the study by Suren, from which we sourced our data (Surén et al., 2013). In the present study, neither socioeconomic status nor access to psychiatric facilities played a determining role in ASD risk. The only factors of consequence were environmental.

Although population density was moderately correlated with dental lead levels (*r* = 0.46), its effects on ASD prevalence diverged in multivariable models. Lead was inversely associated with ASD prevalence, while population density showed a positive association—statistically significant when the outlier county of Telemark was excluded. This suggests that population density is not simply a proxy for lead but rather likely reflects a distinct set of urban-related vehicle emission exposures that may independently increase ASD risk. The association between population density and ASD only emerged after adjusting for lead levels and with incorporation of the later birth cohort, suggesting that lead exposure may suppress the apparent effect of urban-related emissions exposures. This lends further support to the hypothesis that declining lead exposure may have unmasked the harmful effects of vehicle emissions associated with urbanicity on ASD risk.

### Prior studies

A recent systematic review of 37 studies found that more than half reported positive associations between lead exposure and ASD, while none reported an inverse association (Wang et al., 2019). However, these studies relied on postnatal biological matrices—such as hair, blood, and urine—collected from children after diagnosis. Similarly, primary teeth have also been employed to compare metal levels, but results have been inconsistent, ranging from null findings (Abdullah et al., 2012; Adams et al., 2007) to reports of higher fetal and postnatal dental lead in ASD twins compared to their non-ASD siblings (Arora et al., 2017).

Importantly, because these studies inferred lead exposure using matrices collected from children, they may be susceptible to reverse causation. Children with ASD often exhibit altered dental enamel formation, marked by stress-induced accentuated lines (Kurek et al., 2020), and have been reported to have lower bone mineral density (Ekhlaspour et al., 2016). Since bone is a critical repository of lead (Bjørklund et al., 2020), a reduction in bone mass can increase the relative percentage weight of sequestered lead within the skeletal and dental matrix.

Furthermore, decreased bone density may facilitate the redistribution of stored lead back into the bloodstream and peripheral tissues like hair. Such physiological differences suggest that elevated postnatal biomarkers may reflect altered mineral homeostasis and storage dynamics inherent to the disorder rather than the primary etiologic exposure. These systemic alterations likely affect the measured concentrations of not only lead but also other metals, including essential minerals. In contrast, the present study estimates exposure using deciduous teeth collected from children born well before the ASD cohorts under study, reducing the potential for bias arising from physiological alterations inherent to the multi-systemic nature of ASD (Kohane et al., 2012).

While clinical studies often focus on the child’s internal lead burden, our results imply that lead’s primary influence on ASD prevalence may occur via competitive interactions at the maternal-fetal interface. Crucially, this mechanism would be entirely invisible to clinical studies utilizing child biomarkers (e.g., blood or hair), as those matrices reflect the child’s own lead accumulation. This divergence in methodology—external environmental proxies versus internal child biomarkers—is likely the primary reason our findings differ from the existing clinical literature.

To date, few studies have examined maternal lead levels and subsequent ASD risk, largely due to the substantial cohort sizes required to observe sufficient outcomes. To our knowledge, this is the first study that has examined fetal exposure to lead using an external proxy. Interestingly, a recent Norwegian case-control study found lower, though not statistically significant, maternal lead levels among ASD cases compared to controls (Skogheim et al., 2021). However, the authors did not adjust for population density or urbanicity, factors that the present study suggests may confound or modify the relationship between lead exposure and ASD prevalence.

### Potential mechanism

Metal dynamics are key to understanding the putative role of lead in modifying risk of ASD. We have previously suggested that maternal intake of iron may reduce ASD risk not by correcting deficiency *per se*, but by competitively inhibiting absorption of other metals with neurotoxic potential (Grossman, 2017). Iron is known to reduce intestinal absorption and mitochondrial uptake of metals such as manganese and zinc (Arnich et al., 2004; Jouihan et al., 2008; Thompson et al., 2006; Whittaker, 1998). Lead may behave similarly within shared metal-transport pathways. For example, experimental studies demonstrate that lead exposure alters zinc homeostasis, increasing urinary excretion and reducing tissue concentrations in bone and brain (Victery, 1987). The divalent metal transporter-1 (DMT-1), which mediates intestinal absorption of iron and other divalent metals, has been shown to transport lead and is expressed in both placenta and mammary tissue (Bannon et al., 2002; Georgieff et al., 2000). Importantly, DMT-1 activity is dynamically regulated by systemic metal levels, allowing competitive inhibition and hierarchical transport among metals (Meltzer et al., 2010; Shawki et al., 2015).

Within this framework, higher maternal or environmental lead exposure may reduce fetal or early-life bioavailability of other metals implicated in neurodevelopmental toxicity by competing for shared transport mechanisms. At the population level, this could manifest as a suppressor effect, whereby counties with historically higher lead exposure exhibit attenuated associations between urban-related exposures—proxied here by population density—and ASD prevalence. As environmental lead levels declined nationally, this competitive buffering may have weakened, allowing associations between population density–related exposures and ASD prevalence to emerge more clearly in later birth cohorts.

This proposed mechanism is consistent with the ecological and temporal patterns observed in the present study but should be interpreted cautiously. It highlights the possibility that metal–metal interactions and shared transport pathways may influence population-level susceptibility to neurodevelopmental risk factors, warranting further investigation in mechanistically focused and prospective studies.

### Negative control outcomes

The absence of an inverse association for ADHD and CP suggests a degree of specificity in the etiologic pathway of ASD, rather than a generalized neurodevelopmental effect. However, this interpretation warrants caution for two reasons. First, in Norway, ascertainment of CP is high, with 89% of affected children registered by 8 years (Andersen et al., 2020). Given this high level of diagnostic coverage, the null result for CP is likely robust and consistent with the understanding that CP is primarily driven by distinct mechanical or vascular insults (Patel et al., 2024). In contrast, it is estimated that only half of children with ADHD are diagnosed within typical primary care, which may limit the statistical power to detect associations for this specific outcome and increase the risk of Type II error (Duric and Elgen, 2011). ASD diagnoses fall between these two extremes; they are generally ascertained at an early age, with a survey of Norwegian clinics reporting that all children were diagnosed by 71 months (Larsen, 2015). Second, there remains the possibility that to inhibit ADHD via a similar mechanism as ASD, a higher dose of lead is required to mitigate exposure to neurotoxicants causative of the condition. This suggests that while the CP null result is a reliable indicator of etiologic specificity, the ADHD result should be interpreted with caution, as both under-ascertainment and potential differences in dose-sensitivity may mask a similar underlying mechanism. As noted above, the county with the highest level of dental lead (Vest-Agder) also had the lowest ASD and ADHD prevalences.

### Limitations

There are several limitations to this study. First, it is subject to ecological fallacy. Our findings describe associations at the aggregate county level, and we cannot directly infer that individual children with higher lead exposure have a lower risk of ASD; any inference regarding individual risk requires further verification through longitudinal cohort studies. While we have attempted to mitigate this risk by adjusting for population-level variables—such as income and psychiatric service availability—this does not eliminate the possibility of unmeasured confounding or the risk of misattributing group-level trends to individuals. Consequently, these conclusions are strictly applicable to the county-level population in Norway and should be viewed as hypothesis-generating. Second, the sample of 19 counties reduces statistical power and limits the generalizability of the findings to different geographical or temporal contexts. While a significant association was detected, the limited number of counties increases risk of effect size inflation. Third, there was a time lag of up to 15 years between dental lead levels and the birth of the cohort of interest, which may have permitted relative levels of lead exposure across counties to have changed. Finally, though we suggest that increased exposure to pollutants may ultimately enhance risk of ASD, a full exploration is outside the scope of this study. These results should, therefore, be interpreted as hypothesis-generating rather than as evidence of a definitive causal or individual-level relationship.

### Public health implications

These findings raise the possibility that declines in environmental lead may have inadvertently unmasked or exacerbated other ASD-related risk factors. While these results do not call for a reversal of lead remediation efforts, they underscore the need for a shift from single-metal thresholds toward comprehensive metal-mixture monitoring—specifically incorporating potential suppressors like lead or iron into risk assessments of potential toxicants.

Furthermore, due to differential development trajectories and the risk of reverse causation in child biomarkers, policy should prioritize the monitoring of maternal and prenatal exposure profiles. Because biological testing for certain essential metals can be inadequate (O’Neal and Zheng, 2015), proxy measures of environmental exposure—such as population density for vehicle emissions—should be integrated into public health surveillance, particularly when other forms of pollution monitoring—such as PM_2.5_—are lacking. Finally, our results suggest that ecological analyses provide unique value in capturing broad environmental interactions that would be infeasible to track with the same geographic scope at the individual level. Such a scope is essential when studying lead exposure, as localized studies often lack the necessary variance in exposure levels to detect meaningful associations.

## Conclusion

This study identifies a previously unreported inverse association between early-life lead exposure and ASD prevalence at the county level in Norway. These findings suggest that declining environmental lead exposure may have modified population-level susceptibility to ASD, potentially through interactions with other environmental exposures at the individual level. Lead may function as a population-level modifier in metal–metal interactions that influence ASD risk. Larger ecological or prospective studies are needed to confirm these findings and to clarify the role of metal interactions in ASD etiology.

## Synopsis

Study question

Does historical environmental lead (Pb) exposure correlate inversely with autism spectrum disorder (ASD) prevalence across Norwegian counties?

What’s already known

ASD prevalence has risen globally over recent decades, but the causes remain uncertain. Diagnostic changes, increased awareness, and environmental factors have all been implicated. A previous study suggested links between metals and ASD, including a protective association with maternal iron intake, possibly due to competitive inhibition with other unknown metals. Global declines in lead exposure coincide with rising ASD rates, however, this relationship has not been evaluated ecologically.

What this study adds

This Norwegian county-level analysis found higher lead concentrations in deciduous teeth were significantly associated with lower ASD prevalence. The results suggest declining environmental lead may have inadvertently heightened ASD risk by unmasking harmful effects of other environmental metals or urban exposures.

## Data Availability

The original contributions presented in this study are included in this article/Supplementary material, further inquiries can be directed to the corresponding author.
