# Supplementary material for: Association between historical lead exposure, population density, and autism prevalence: a county-level ecological study in Norway
Source: Front Neurosci. 2026 Feb 4;20:1729731. doi: 10.3389/fnins.2026.1729731 (PMC12913441; doi:10.3389/fnins.2026.1729731)
Supplement: Supplementary file 1 [file Data_Sheet_1.docx]

**Supplemental Table 1**. Spearman correlation matrix of covariates.

|  | Lead | Pop. Density | Income | Persons / psychiatric unit |
| --- | --- | --- | --- | --- |
| Lead | 1 | **0.46** | 0.13 | 0.1 |
| Pop. Density | - | 1 | **0.54** | -0.03 |
| Income | - | - | 1 | -0.32 |
| Persons / psychiatric unit | - | - | - | 1 |

Note: Bold p-values indicate statistical significance at α = 0.05.

**Supplemental Table 2**. Sensitivity regression analysis of natural log-transformed ASD prevalence by ±1 standard deviation of geometric mean dental lead levels at county level

| **Model** | **Variable** | **Coefficient** | **SE** | **p-value** |
| --- | --- | --- | --- | --- |
| **+1 STD** | ln(Density) | 0.10275 | 0.04878 | 0.0513 |
|  | Lead | -0.47944 | 0.13236 | **0.0023** |
|  | Model Adj. R-Sq. | 0.3946 (p = **0.0070**) | | |
| **-1 STD** | ln(Density) | 0.06341 | 0.05399 | 0.2574 |
|  | Lead | -1.45240 | 0.62603 | **0.0339** |
|  | Model Adj. R-Sq. | 0.1756 (p = 0.0832) | | |

Bold p-values indicate statistical significance at α = 0.05. lead = dental lead concentration. Each model includes ln population density as a covariate along with dental lead concentrations.


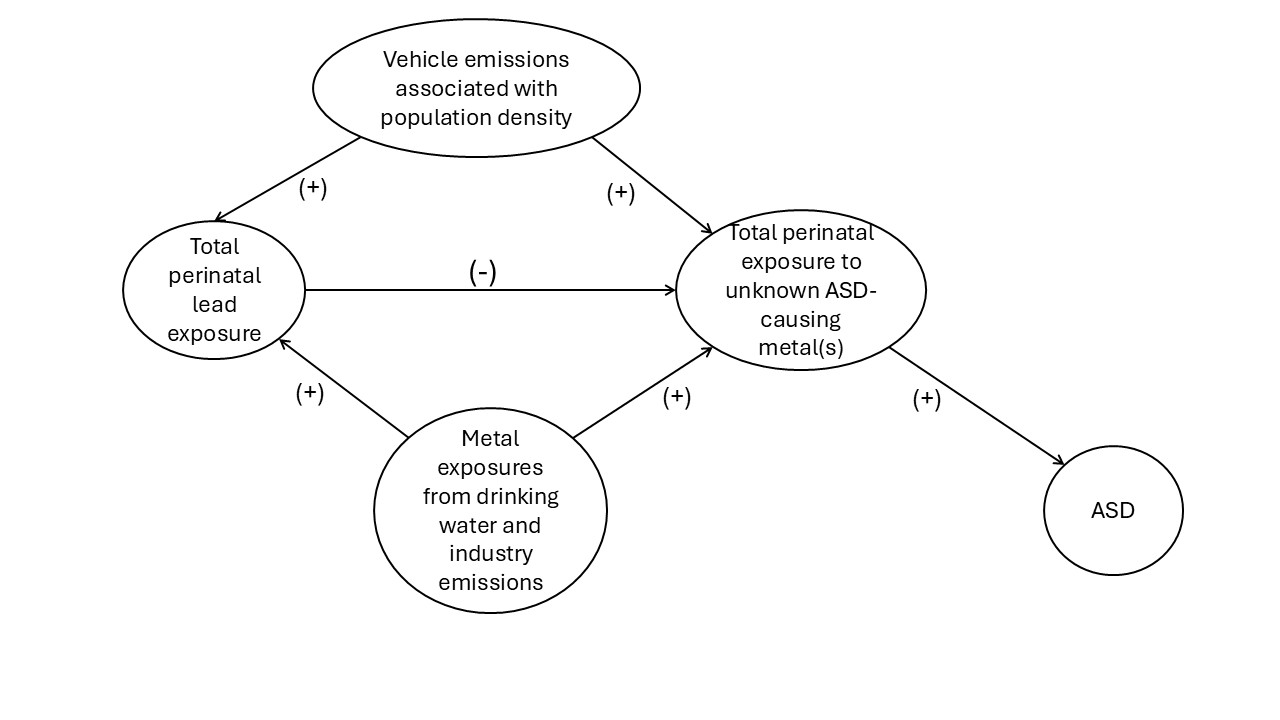


**Figure 1S.** Directed acyclic graph (DAG) illustrating hypothesized relationships between lead exposure, other unknown metal exposures, and autism spectrum disorder (ASD) risk. Population density is assumed to increase exposure to lead and one or more unknown ASD-causing metal pollutants. Lead exposure is hypothesized to reduce this toxic metal burden—via competitive inhibition mechanisms—thereby lowering ASD risk. This indirect pathway supports lead’s role as a suppressor variable in the relationship between population density and ASD prevalence.

**Figure 2S.** Added-variable plot showing the adjusted association between pb and log(ASD) after controlling for log(density). One county (Telemark) appears as a potential outlier.
